# Hospitalized Children: Anxiety, Coping Strategies, and Pretend Play

**DOI:** 10.3389/fpubh.2019.00250

**Published:** 2019-09-06

**Authors:** Elisa Delvecchio, Silvia Salcuni, Adriana Lis, Alessandro Germani, Daniela Di Riso

**Affiliations:** ^1^Dipartimento di Filosofia, Scienze Sociali, Umane e della Formazione, Università di Perugia, Perugia, Italy; ^2^DPSS – Dipartimento di Psicologia dello Sviluppo e della Socializzazione, Università di Padova, Padova, Italy

**Keywords:** anxiety, coping, symbolic play, hospitalized child, assessment

## Abstract

The aim of this paper was to assess strengths and fragilities in children aged 6 to 10 who suffered one or more hospitalizations. State and trait anxiety, coping abilities, and cognitive and affective functioning through play were assessed using a triangulation approach. Fifty hospitalized children aged 6–10 were compared to 50 non-hospitalized children, and children at first admission were compared with children with more than one hospitalization experience. The State-Trait Anxiety Scales Inventory for Children was administered for assessing trait and state anxiety, and the Children's Coping Strategies Checklist (Revision 1) was administered to assess coping dimensions. The Affect in Play Scale - Preschool - Brief (Extended version) was used to assess cognitive and affective dimensions of play. No significant differences were found for trait anxiety between hospitalized vs. non-hospitalized children. Instead, as expected, state anxiety was significantly higher in hospitalized childen than in the non-hospitalized children. Hospitalized children reported higher scores than non-hospitalized children in support-seeking strategies. As for pretend play, hospitalized children showed significantly higher cognitive scores than non-hospitalized children. However, hospitalized children appeared significantly more restricted in their affect expressions. No significant differences were found for play and anxiety scores between children admitted for the first time in the hospital ward and children with more than one admission. However, children at first admission scored higher in coping and positive cognitive restructuring and in avoidance-coping strategies than children with more than one admission. The initial assessment of the interplay of key variables such as anxiety, coping and play can inform healthcare professionals by serving as a guide in order to determine a child's risk for negative psychological outcomes due to hospitalization, to plan appropriate interventions and to provide substantial assistance to hospitalized children in the future.

## Introduction

Hospitalization for children means leaving their home and their caregivers and siblings and an interruption of their daily activities and routines. Moreover, hospital wards are often associated with staying in a “cold and medical” setting, facing fear of medical examinations, pain, uncertainty, and loss of control and safeness [e.g., (1, 2)]. This is particularly true for elementary school children who are involved in mental, emotional, and social adjustment developmental tasks. Literature about hospitalization during childhood underscores how, in the short term, extreme distress may compromise the completion of a required medical procedure, while in the long term it may lead to difficulties in future intakes that discourage the use of medical treatments (3–6). Moreover, anxiety-provoking experiences (such as hospitalizations) can affect children physical growth, personality, or emotional development (7). Burns-Nader and Hernandez-Reif (2) stressed that to determine children's needs in the medical setting, specialists have to carry out a psychological assessment in order to detect potential stress, anxiety, coping abilities, and play skills to provide age-appropriate interventions.

Usually, children feel anxious before encountering medical professionals, as well as experiencing a hospitalization (7). Empirical studies suggest that children express anxiety through regression in behaviors, aggression, lack of cooperation, withdrawal, and difficulty recovering from procedures (8, 9). Literature shows that children involved in psychological programs were more able to contain anxiety, showing lower levels of anxiety assessed before surgery, and reporting less postoperative anxiety (10). Previous studies supported the importance of specific clinical measures to assess children's anxiety in medical settings (11). Indications provided by tailored tools, might be helpful to support children in approaching medical situations with a sense of comfort, achievement, and control. Few empirical studies have been carried out on levels of trait and state anxiety in hospitalized children (12). Trait anxiety follows the child in everyday experiences including hospitalization and as such, if elevated, has to be recognized as a vulnerability for the child. State anxiety could originate from the hospital experience. The literature shows that among children aged 5–11, it vanishes from hospital admission to discharge (13). Trait vs. state anxiety is not often assessed, and subsequently undertreated (14). Trait anxiety plays an important role in the child's response to hospitalization (9, 15). The higher a child's trait anxiety, the higher his or her perception of hospitalization as a stressful experience will be and the less effective will be his or her ability to cope (15, 16).

Burns-Nader and Hernandez-Reif (2) suggested it is fundamental to foster effective coping to minimize anxiety in children experiencing a medical situation. Coping in children can be defined as a collection of conscious and purposeful efforts that are directed at the regulation of aspects of the self (emotion, cognition, behavior, and physiology) and the environment in contexts involving stress [e.g., (17–19)]. Adaptive coping strategies could fail under stressful conditions (20–22). Effective coping behaviors provide resilience to mitigate the likelihood of adverse outcomes and potentially enhance growth (23–26). Effective coping promotes adjustment to stressful life events, well-being, competence and resilience during childhood and adolescence (27). Blount et al. (28) highlighted the importance to consider coping a multidimensional construct. Specifically, Skinner et al. (21) suggested that five categories of coping are clearly crucial across ages and have been empirically supported in children and adolescents (17, 29–32): problem-solving, positive cognitive restructuring (active coping), support seeking, avoidance, and distraction. Research findings suggested that psychological outcomes related to hospitalization are linked to children's coping styles (33). Avoidant coping is mainly used during the acute phase of health care or hospitalization, whereas active coping is prominent in the recovery phase (7). Avoidant coping strategies are characterized by restricted thoughts on an upcoming event, denial of worries, and disconnection from stressful stimuli. They seem to be less effective in reducing the stress connected to hospitalization (12). With regard to the link between previous hospitalization and anxiety/coping, conclusions are not well-established. Some research has found that previous hospitalization is not related to a child's anxiety or coping (12, 34). However, children with no previous hospitalization, as well as those with fewer previous surgeries, showed higher anxiety than the ones who were already familiar with the medical setting (13, 35).

Among others, play is considered a coping method for children who experience a hospitalization, because play activity allows to express and elaborate affects and to show problem-solving abilities (36, 37). Play allows children to convey their feelings and control stressful experience because through it children can recreate and transform their life events (2, 38, 39). In a study in which outcome measures were not assessed, hospitalized children stated that they used play to manage stressful experiences more frequently than non-hospitalized children (2, 40). For such a purpose, symbolic play, or pretend play, represents an important integration opportunity of cognitive, affective, and interpersonal competencies. Play facilitates representation of the world and helps children to express their feelings, make choices, transform stories, use imagination, focus on stressful or unfamiliar themes, and develop skills (41–44). A growing amount of research has supported the validity and reliability of the Affect in Play Scale [APS, (43)], a measure to assess pretend play with children. Both the original and the brief version, which does not include video-recording, showed good psychometric properties in school and preschool-based samples of typically developing children in the United States and in Italy (45–52). The existing literature underlines the importance of providing children with play sessions in the hospital playroom, at the bedside, or even in waiting rooms of hospital wards (2). Li et al. (53) highlighted the role of play intervention in reducing distress and anxiety in children that are hospitalized. Although, O'Connor (54) indicated pretend play as a natural mediator with hospitalized children, there is a paucity of valid and reliable tools devoted to it (55). So, the assessment of cognitive and affective abilities in pretend play during hospitalization of children, should be seen as beneficial for researchers and clinicians (56, 57).

The aim of the current paper was to assess the strengths and fragilities of hospitalized children aged 6 to 10 who suffered one or more hospitalization, comparing them to a community sample of non-hospitalized children. More specifically, the purposes of the current paper were twofold: to compare the level of state and trait anxiety, coping, and pretend play in (a) hospitalized vs. non-hospitalized children and (b) children at first admission vs. children with more than one hospitalization experience. In order to accomplish these goals, state and trait anxiety, coping abilities, and cognitive and affective functioning through play were assessed using a triangulation approach, which refers to the application and combination of several research methods in the study of the same phenomenon (58–60). In this study different information about the hospitalized children was collected using quantitative mixed methods (questionnaires and play tasks) gathered by the children themselves and compared with the same tools gathered by non-hospitalized children.

Attention was given to tools with adequate psychometric properties that can inform about a child's life by serving as a guide for initial assessments in pediatric wards where often a qualitative assessment is preferred. We hypothesized no significant differences in trait anxiety between hospitalized and non-hospitalized children, because it accompanies the child in everyday experience. Instead, state anxiety was expected to be higher.

## Materials and Methods

### Procedure

The administration was carried out in compliance with the ethical standards for research outlined in the Ethical Principles of Psychologists and Code of Conduct (61). The study was approved by the ethics committee of the hospital including the pediatric unit and by the ethics committee for psychological research of Padova University (2017/num 2310). Each participant was met individually in a place where he or she could comfortably play and complete the questionnaires. During each session, participants were first engaged in the play task to assess cognitive and affective pretend play processes and later the two questionnaires were administered. No reward was offered for participation.

### Participants

Power analysis to estimate the sample size was carried out using G^*^Power 3.1 (62). The sample size was inferred by considering three factors: a significance level of 0.05 (one tail); a medium effect size based on previous studies (53); and a power of 0.80. Power analysis indicated that there was an 80% chance of correctly rejecting the null hypothesis of no difference between hospitalized and non-hospitalized children, with a total sample of 100 (50 + 50) participants.

Thus, 50 hospitalized children (22 boys and 28 girls) aged 6–10 were recruited from a pediatric clinic in Northern Italy and 50 non-hospitalized children (22 boys and 28 girls) were recruited from elementary schools in Northern Italy.

#### Hospitalized Children

Participants were a convenient sample of children admitted at the Pediatric Clinic of the University of Padova, during a 13-month period. In this period, 50 pediatric patients met the criteria selected for the present research. Inclusion criteria included children diagnosed by a physician as affected by middle (e.g., rheumatologic, cardiac, and metabolic pathologies) or transient pathologies (e.g., appendicitis or tonsillitis) or both. Moreover, children with psychiatric symptoms, severe cognitive impairment, and maladjustment were excluded. This information was collected in an anamnestic form fulfilled by parents, who signed written consent. Forty-two percent of selected children (*n* = 21) were at their first admission into the ward, and 58% (*n* = 29) had more than one admission to the hospital ward. Among the latter, 11% (*n* = 5) were admitted for different reasons, whereas 49% (*n* = 24) were admitted for the same reason. The admission period lasted between 5 and 10 days. Measures were administered in a quiet room, after a warm-up meeting with the examiner. The administration was scheduled in order not to interfere with the daily medical routine.

#### Non-hospitalized Children

Non-hospitalized children were selected randomly from a larger sample matched by gender and age with the hospitalized children. Children with psychiatric symptoms, severe cognitive impairment, and maladjustment were excluded. Consent forms were sent home to parents. Children were allowed to participate in the study after parents provided written consent. A brief questionnaire about the children's physical health was sent to parents for the assessment of possible hospitalization. Each participant was met individually during school hours in a room where the children could comfortably play and complete the questionnaires. Some familiarity with the examiner was established before task administration. During each session, participants were first engaged in the play task to assess cognitive and affective pretend play processes and later the two questionnaires were administered.

## Measures

State-Trait Anxiety Inventory for Children [STAI-C; (63, 64)] is a self-report measure developmentally adequate for assessing anxiety symptoms in children aged 9–12 years, but it can be used with younger children with average or above average reading abilities. It includes two separate scales for measuring two anxiety concepts: state and trait anxiety. The state scale, a measure of transitory anxiety states, consists of 20 statements that ask children how they feel at a particular moment in time. The items all start with the stem “I feel” and next to each stem respondents have to choose among three responses the one that best describes their state (e.g., very calm, calm, or not calm). The trait scale consists of 20 statements that ask children how they generally feel. It measures relatively stable individual differences in anxiety proneness. The items are rated on a 3-point scale with responses: *hardly-ever, sometimes*, and *often*. STAI-C showed adequate psychometric features in both international and national samples [e.g., (65, 66)]. Cronbach's alpha for the state scale was 0.79 for hospitalized and 0.71 for non-hospitalized children; alphas for the trait scale were 0.77 and 0.76 for hospitalized and non-hospitalized children, respectively.

Children's Coping Strategies Checklist-Revision 1 [CCSC-R1; (67)] includes 54 statements. Each statement starts with “If I have a problem” and is followed, for example, by “I tell others how I would like to solve it.” Children have to indicate how frequently they usually adopted the coping strategies described in the item on a 4-point Likert scale: 1 = *never*, 2 = *sometimes*, 3 = *often*, and 4 = *always*. CCSC-R1 is composed of 13 subscales and five dimensions: problem focused, coping and positive cognitive restructuring, distraction coping strategies, avoidance coping strategies, and support-seeking strategies. Examples of items are as follow: problem focused (“You thought about what you needed to know before”); coping and positive cognitive restructuring (“You told yourself you could handle whatever happens”); distraction coping strategies (“You watched TV”); avoidance coping strategies (“You tried to stay away from things that made you feel upset”); support-seeking strategies (“You talked to someone who could help you solve the problem”). Thus, CCSC-R1 includes two dimensions of active coping (problem-focused coping and positive cognitive restructuring), two dimensions connected with avoidance (distraction and avoidance coping strategies), and finally one dimension connected with support-seeking strategies. In this paper, each scale was made of the sum of the items. In the Italian validation, all dimensions yielded adequate reliability (68). Cronbach's alphas for the current study ranged from 0.67 to 0.87 for hospitalized children and from 0.55 to 0.77 for non-hospitalized ones.

The extended version of the Affect in Play Scale-Preschool Brief Version [APS-P-BR; (43, 46–50)] is a structured individually administered 5 min play task that allows evaluation of the affective and cognitive aspects (affect, imagination, organization, and comfort) in child's play using a standardized and empirically validated administration procedure and *in vivo* scoring attribution (43). Children are asked to play with a set of plastic and stuffed toys [for further detail see (49)]. Six primary scores (four cognitive and two affective) are assigned using a detailed scoring manual (43). The four cognitive scores are organization, elaboration, imagination, and comfort, coded on a 4-point Likert scale. Two main scores concerning affects are frequency of affect and tone [see (49)]. Psychometric characteristics of APS-P-BR Extended version showed satisfactory results (49).

## Data Analysis

Student's one-tailed *t*-tests for independent samples was performed on the APS-P-BR Extended version, state and trait STAI-C, and CCSC-R1 scores to compare hospitalized vs. non-hospitalized children. Moreover, a Student's one-tailed *t*-test for independent samples on all variables was performed to compare means of children who were admitted in the hospital ward for the first time vs. children who were admitted more than one time. A one-tailed test was considered appropriate because the aim was to check if the estimated value may depart from the reference value in only one direction.

## Results

Means and standard deviations for all variables for hospitalized and non-hospitalized children are reported in Table 1.

**Table 1:** Means, standard deviations and Student's *t*-test for hospitalized and non-hospitalized children.

	**Hospitalized (*****n*** **=** **50)**		**Non-hospitalized (*****n*** **=** **50)**		***t*_**(98)**_**	p^*^	***d***
	**M**	**SD**	**M**	**SD**			
Age	8.10	1.62	8.96	0.98			
**STAI-C**							
State	31.22	6.76	29.34	4.15	1.68	<0.050	0.33
Trait	36.98	6.44	35.98	6.19	0.79	0.215	0.16
**CCSC-R1**							
Problem focused	2.48	0.54	2.64	0.49	−1.53	0.064	0.31
Positive cognitive restructuring	2.54	0.57	2.51	0.45	0.34	0.367	0.06
Distraction	2.64	0.71	2.87	0.52	−1.85	<0.050	0.37
Avoidance	2.65	0.50	2.50	0.72	1.60	0.056	0.24
Support–seeking	2.50	0.72	2.15	0.54	2.79	<0.010	0.55
**APS-P-BR**							
Organization	3.36	0.85	3.18	0.83	1.07	0.143	0.21
Elaboration	3.24	0.87	2.48	0.81	4.51	<0.010	0.90
Imagination	3.42	0.76	2.96	0.70	3.15	<0.010	0.63
Comfort	3.28	0.86	3.14	0.88	0.81	0.211	0.16
Frequency of affect	3.52	0.74	3.94	0.24	−3.15	<0.010	0.76
Tone	2.74	0.83	2.96	0.40	−1.69	<0.050	0.34

* *one-tailed*.

Student's *t*-test value for independent samples was calculated for all tools administered to compare hospitalized vs. non-hospitalized children. Results are shown in Table 1.

Regarding anxiety, as expected no significant differences were found for trait anxiety. However, hospitalized children showed a higher level of state anxiety with a medium effect size. Focusing on coping strategies, support-seeking strategies showed hospitalized children reporting higher scores than non-hospitalized ones, with medium effect size. In regards to distraction, hospitalized children reported lower distraction scores. No other differences were found concerning this measure. As for pretend play, hospitalized children showed significantly higher elaboration and imagination than non-hospitalized children, with high and medium effect size, respectively. However, they appeared significantly more restricted in their affect expressions and with lower scores on tone, with high and medium effect size.

Means and standard deviations for children admitted for the first time in the hospital ward and children with more than one admission as well as Student's *t*-test for independent samples are reported in Table 2.

**Table 2:** Student's t for hospitalized children with one or more admissions.

	**First admission (*****n*** **=** **21)**		**More than one (*****n*** **=** **29)**		***t*_**(48)**_**	p^*^	***d***
	**M**	**SD**	**M**	**SD**			
**STAI-C**							
State	31.62	7.18	30.93	6.56	0.35	0.363	0.10
Trait	35.91	6.12	37.76	6.67	−1.00	0.160	0.29
**CCSC-R1**							
Problem focused	2.59	0.69	2.40	0.40	1.21	0.115	0.37
Positive cognitive restructuring	2.75	0.50	2.39	0.57	2.29	<0.050	0.67
Distraction	2.69	0.54	2.60	0.82	0.46	0.323	0.13
Avoidance	2.81	0.52	2.53	0.47	1.96	<0.050	0.56
Support–seeking	2.55	0.77	2.47	0.70	0.41	0.343	0.11
**APS-P-BR**							
Organization	3.33	0.86	3.38	0.86	−0.19	0.426	0.06
Elaboration	3.19	0.86	3.28	0.88	−0.34	0.368	0.10
Imagination	3.33	0.80	3.48	0.74	−0.68	0.248	0.19
Comfort	3.29	0.78	3.28	0.92	0.04	0.484	0.01
Frequency of affect	3.48	0.68	3.55	0.78	−0.36	0.362	0.09
Tone	2.90	0.94	2.62	0.73	1.20	0.117	0.33

* *one–tailed*.

No significant differences were found for play and anxiety scores. For coping strategies, children at first admission scored higher in coping and positive cognitive restructuring and in avoidance-coping strategies than children with more than one admission. Effect sizes of these differences were medium.

## Discussion

This triangulation study evaluated state and trait anxiety, coping, and pretend play in a sample of hospitalized school-age Italian children compared with a control group of children of the same age never hospitalized. Trait anxiety did not differentiate significantly hospitalized vs. not hospitalized children, meaning that anxiety levels that typically accompany children during their everyday life experiences did not seem to be affected by the hospitalization. Trait anxiety did not differentiate significantly children at their first admission vs. children who already experienced hospitalization, meaning that the structural level of anxiety, so-called trait anxiety, was maintained at a normative level and was not undermined by the hospitalization experience (9, 15). As expected, state anxiety that was influenced by stressful transient experiences, such as the hospitalization, was higher in hospitalized children, with no difference in one-admission or multiple-admission subgroups (53). Referring to coping, hospitalized children reported a higher level of support seeking but lower score on distraction. As expected, children in the hospital ward are looking for more support by parents, nurses, or volunteers, but they are forced to reduce distraction strategies, such as sport or watching TV. No significant differences were found between hospitalized and non-hospitalized children for the two dimensions of active coping—problem-focused and positive cognitive restructuring. Literature suggested that an increase in problem-solving strategies is typical of this stage of development (27), showing that school age children are involved in a gradual shift from behavioral actions to more cognitive-based coping (69, 70). This pattern seemed to be valid independently from hospitalization experience. However, when looking at the two subsamples of hospitalized children, positive cognitive structuring and avoidance appeared significantly higher for children in their first admission. As Wilcox (33) suggested, the effectiveness of coping strategies are affected by recurrence and length of admissions. Children with more than one hospitalization are less prone in avoiding the stress of the situation and in recalling positive thoughts. Despite the unpleasant experience of hospitalization, hospitalized children in this study were able to maintain an organized pretend play and appeared comfortable in play at the same level as non-hospitalized children. Moreover, they used a higher amount of variety and complexity of embellishment in the story themes (elaboration) and a higher amount of fantasy and number of transformations (e.g., using one thing as another) in the play (imagination). Their more sophisticated elaboration of the scenario and the more prominent use of transformation in their storytelling might represent a useful way to deal with the distressing, unfamiliar, and painful reality of the hospitalization experience [e.g., (54)]. However, hospitalized children were more restricted in their expressions of affections in play, maybe for fear of being overwhelmed by a great variety and amount of affects that hospitalization could activate. It is interesting that the trend was maintained both for children at first hospitalization as well as for children with more than one hospitalization. Repeated hospitalizations do not seem to influence cognitive or affective components of play. Altogether, in this study the results showed that hospitalized children were able to organize a pretend play and trait anxiety did not differ from not clinical children. Moreover, they expressed active coping, but they also try to use avoidance defenses and they recognized their need for support.

However, this research has several limitations. This study was exploratory in nature. First, the sample was small and was made up of children affected by different kinds of diseases. Moreover, the generalizability of the results might be biased by the sampling method used for the collection of hospitalized children. Even though the sample size was supported by the power analysis, the number of participants was also affected by the recruitment in a hospital ward and by the restricted time schedule of the agreement with the hospital itself. Research and clinical literature showed the use of play in hospital, but often introduced and interpreted in a qualitative way: the use of the APS-P-BR Extended version would give the experts a way to assess in a more empirical way how the hospitalized children would be able to organize or not a pretend play in a distressful period of their lives.

There is a paucity of research on quantitative assessment with a triangulation method, mostly used to combine qualitative and quantitative approaches. The present paper aimed to propose the use of three validated measures to highlight children functioning in the experience of hospitalization. Even though anxiety, coping, and play are singular important aspects, their interplay might shed more light on the way children face a stressful experience, capturing the different dimensions of the same phenomenon. Referring to Lewick (7), beginning a health care assessment as it was proposed in this study stressing children's resources and labilities means to recognize and support patients' resilience, or strengths, and contributes in understanding the way in which a child patient can manage struggles in his or her life. In this way, a medical professional helps the patient to focus on and bring out his or her internal resources in order to deal with and overcome his or her concerns about the medical problems. Both strengths and difficulties should be relevant for—and emphasized by—medical professionals. In addition, starting from the resources and reframing negative talk around the child, a health care provider can decrease a child's anxiety and maladaptive trauma responses, regardless of the specific reasons for medical treatment. At last, the advice given to medical professionals to speak aloud a child's positive qualities during the first assessment (as well as whenever possible) is of crucial importance because it may be the only time in a day a child hears about them.

## Data Availability

The datasets for this manuscript are not publicly available because Data available upon request. Requests to access the datasets should be directed to elisa.delvecchio@unipg.it.

## Ethics Statement

This study was carried out in accordance with the recommendations of the Ethical Principles of Psychologists and Code of Conduct (61) with written informed consent from all subjects. All subjects gave written informed consent in accordance with the Declaration of Helsinki. The protocol was approved by the Ethic committee of the hospital including the pediatric unit and by the Ethic committee for psychological research of Padova University (#2310).

## Author Contributions

AL, DD, ED, and SS contributed conception and design of the study. AG organized the database and performed the statistical analysis. AL wrote the first draft of the manuscript. DD, ED, and SS wrote sections of the manuscript. All authors contributed to manuscript revision, read, and approved the submitted version.

### Conflict of Interest Statement

The authors declare that the research was conducted in the absence of any commercial or financial relationships that could be construed as a potential conflict of interest.
